# Carbon Dioxide and Fruit Odor Transduction in *Drosophila* Olfactory Neurons. What Controls their Dynamic Properties?

**DOI:** 10.1371/journal.pone.0086347

**Published:** 2014-01-21

**Authors:** Andrew S. French, Shannon Meisner, Chih-Ying Su, Päivi H. Torkkeli

**Affiliations:** 1 Department of Physiology and Biophysics, Dalhousie University, Halifax, Nova Scotia, Canada; 2 Neurobiology Section, Division of Biological Sciences, University of California San Diego, La Jolla, California, United States of America; AgroParisTech, France

## Abstract

We measured frequency response functions between odorants and action potentials in two types of neurons in *Drosophila* antennal basiconic sensilla. CO_2_ was used to stimulate ab1C neurons, and the fruit odor ethyl butyrate was used to stimulate ab3A neurons. We also measured frequency response functions for light-induced action potential responses from transgenic flies expressing H134R-channelrhodopsin-2 (ChR2) in the ab1C and ab3A neurons. Frequency response functions for all stimulation methods were well-fitted by a band-pass filter function with two time constants that determined the lower and upper frequency limits of the response. Low frequency time constants were the same in each type of neuron, independent of stimulus method, but varied between neuron types. High frequency time constants were significantly slower with ethyl butyrate stimulation than light or CO_2_ stimulation. In spite of these quantitative differences, there were strong similarities in the form and frequency ranges of all responses. Since light-activated ChR2 depolarizes neurons directly, rather than through a chemoreceptor mechanism, these data suggest that low frequency dynamic properties of *Drosophila* olfactory sensilla are dominated by neuron-specific ionic processes during action potential production. In contrast, high frequency dynamics are limited by processes associated with earlier steps in odor transduction, and CO_2_ is detected more rapidly than fruit odor.

## Introduction

Carbon dioxide sensitivity occurs in a variety of insects, including some with major health and agricultural impacts on humans. In *Drosophila* antennae, one of the four neurons (ab1C) of the largest basiconic sensilla (ab1) responds to CO_2_, whereas other neurons in these sensilla respond to fruit odors. The ab1C neurons express two gustatory receptors GR21a and GR63a that together comprise the CO_2_ receptor [1], [2]. These neurons lack the odorant receptors (ORs) and the auxiliary OR83b (Orco) receptors, which are common to all other neurons that mediate odor responses in basiconic sensilla [3], [4], [5].

*Drosophila* also possess a family of ionotropic chemoreceptor molecules, located in coeloconic sensilla and other antennal structures [5], [6], which include acid sensitive receptors responsive to high concentrations of CO_2_ [7]. Behavioral responses to CO_2_ are correspondingly complex. CO_2_ alone may be attractive or repellent under different testing conditions [1], [8], [9], [10] while combination of CO_2_ with other odors may overcome repulsion or create attraction [8], [9], [11].

Time dependence of odorant response is crucial for many olfactory functions, but relatively poorly understood. Moths and bees are sensitive to the temporal structures of odorants, and mosquitoes to CO_2_ plumes [12], [13], [14], [15], [16]. Other hematophagous insects are attracted to CO_2_ pulsations in the human breathing range [17]. Dynamic input-output characterization can identify time-dependent behavioral limitations and may also help to identify physiological mechanisms, as has been shown in a range of sensory receptors [18], [19], [20].

We previously showed that frequency responses of basiconic sensilla neurons to fruit odors could be well-fitted by simple band-pass filter functions, with the response declining at both extreme low and high frequencies. This characterization applied to both excitatory and inhibitory odor-sensillum combinations [21].

Here, we developed an approach to test and characterize the dynamic responses of ab1C neurons to CO_2_. For comparison to olfactory transduction in other basiconic sensilla we also used the same apparatus to measure frequency responses in odorant sensing ab3A neurons that were stimulated by ethyl butyrate. In theory, overall sensory neuron dynamics could be controlled at several different stages of the mechanism between odorant arrival and action potential production. To separate these dynamic contributions we measured frequency responses between light stimulation and action potentials in transgenic flies expressing H134R-Channelrhodopsin-2 (ChR2) in ab1C and ab3A neurons [11], for direct comparison with chemical detection in the same neuron types. Our results indicate that low frequency sensitivity varies with neuron type, and is dominated by processes associated with action potential production. In contrast, high frequency sensitivity is probably limited by early stages of odor transduction, with CO_2_ providing a more rapid response than fruit odors.

## Materials and Methods

### Ethics Statement

Cold anesthesia was used prior to each experiment. All procedures followed a protocol (I12-29) approved by the Dalhousie University Committee on Laboratory Animals.

### Preparation and Electrophysiology

Wild type flies, *Drosophila melanogaster*, Oregon R #2376 (Bloomington *Drosophila* stock center, Bloomington, IN) were raised and maintained in an incubator using a standard diet [22] at a temperature of 23°C under a 13 hour light/11 hour dark cycle. For optogenetic experiments, Channelrhodopsin-2 (H134R-ChR2) was expressed in ab1C sensilla that also express the GR21a/GR63a gustatory receptors and ab3A sensilla that also express the OR22a olfactory receptor. ChR2 flies were reared in constant darkness on fly food supplemented with 100 µM all-*trans* retinal [11]. Flies of either sex were used within two days of hatching.

Flies were located in the cut end of a 100 µl plastic pipet tip. Tungsten electrodes were fabricated from 0.1 mm diameter wire, sharpened electrolytically by passing current through the tip into concentrated potassium hydroxide solution, and pushed into the sockets of basiconic sensilla located near the proximal medial border of the posterior surface of the third antennal segment. A reference tungsten electrode was inserted into the contralateral eye. Single unit recordings were fed to a Grass P55 amplifier (Grass Technologies, West Warwick, RI).

### Olfactory Stimulation

**Figure 1:**
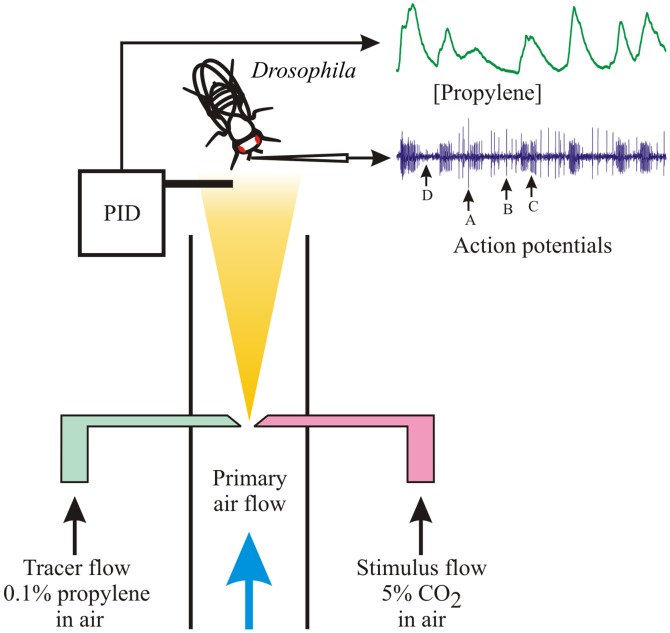
Stimulation of *Drosophila* antenna by randomly varying CO_2_ concentration. Primary air was driven by a fan through a 20 mm diameter, circular flow tube made from fluorinated ethylene propylene. CO_2_ was released into the laminar flow from a plastic pipet tip. A random binary sequence drove a servo-controlled loudspeaker to move a silicone bead against the tip end, alternately starting and stopping the flow of CO_2_ into the stream. This resulted in a randomly varying, wide bandwidth concentration of CO_2_ at the tube mouth. The fly was held in the center of the tube, within 5 mm of its mouth. CO_2_ concentration at the antenna was estimated by a surrogate tracer gas, propylene (0.1% in air), released from an identical pipet tip and occluded by the same silicone bead. Propylene concentration was measured by a miniature photoionization detector located within 1 mm of the antenna. Tungsten electrodes recorded action potentials from single antennal basiconic sensilla. Traces show PID signal and action potentials during CO_2_ stimulation, with ab1A-ab1D neuron action potentials indicated. Ethyl butyrate stimulation used the same apparatus, with the odorant placed in a filter paper cartridge in series with the air/propylene stream, and no CO_2_.

The stimulating system (Fig. 1) was developed from methods described previously [21], [23], [24]. A fan created laminar airflow through a 120 mm long, 20 mm diameter tube. The fly was positioned within 2–3 mm of the exit and 2–3 mm of the tube center line. Secondary gas flow into the primary air flow came from two identical plastic 200 µl pipet tips (Progene, St. Laurent, PQ, Canada). One tip was fed by 0.1% propylene in air at 50 kPa, the other by 5% CO_2_ in air at 50 kPa. Flow from both pipet tips was switched on or off simultaneously by occlusion with a silicone plug driven by a servo-controlled loudspeaker. The same stimulating system was used for ethyl butyrate experiments, but the CO_2_ source was disconnected and an odorant cartridge made from the shaft of a 5 ml transfer pipet containing a rectangular piece of filter paper (45 mm×15 mm) was inserted into the propylene/air feed. Ethyl butyrate (0.1% v/v in mineral oil) was loaded in 100 µl volumes onto the filter paper cartridge.

Propylene concentration at the fly antenna was measured by a miniature photoionization detector (mini-PID, Model 200A, Aurora Scientific Inc, Aurora, ON, Canada). The tip of the inlet probe was located directly above and within 2 mm of the antenna. The PID frequency response was 0–330 Hz and its concentration range was 0.05–500 ppm propylene.

All experiments were performed at room temperature (20±2°C) in a controlled humidity chamber (<40%). The fly preparation was mounted on an air driven anti-vibration table. The stimulating system was mounted separately, and mechanically isolated from the fly. All chemicals were purchased from Sigma (Oakville, ON, Canada) and gasses from Linde (Dartmouth, NS, Canada).

### Optical Stimulation

A high intensity light emitting diode (LED, V Star LXHL-LB5C, peak emission 470 nm, Luxeon, San Jose, CA, USA) was driven by a custom built, linear voltage to controlled current power supply. The M-sequence signal was filtered by a nine-pole, active 100 Hz low-pass filter before driving the LED, to limit the upper signal bandwidth to a similar frequency as the olfactory stimulation system, and also to satisfy the Nyquist sampling criterion [25]. LEDs were optically coupled to a fiber optic light guide with the tip located within 1–2 mm of the sensillum being recorded.

### Experimental Control and Data Processing

**Figure 2:**
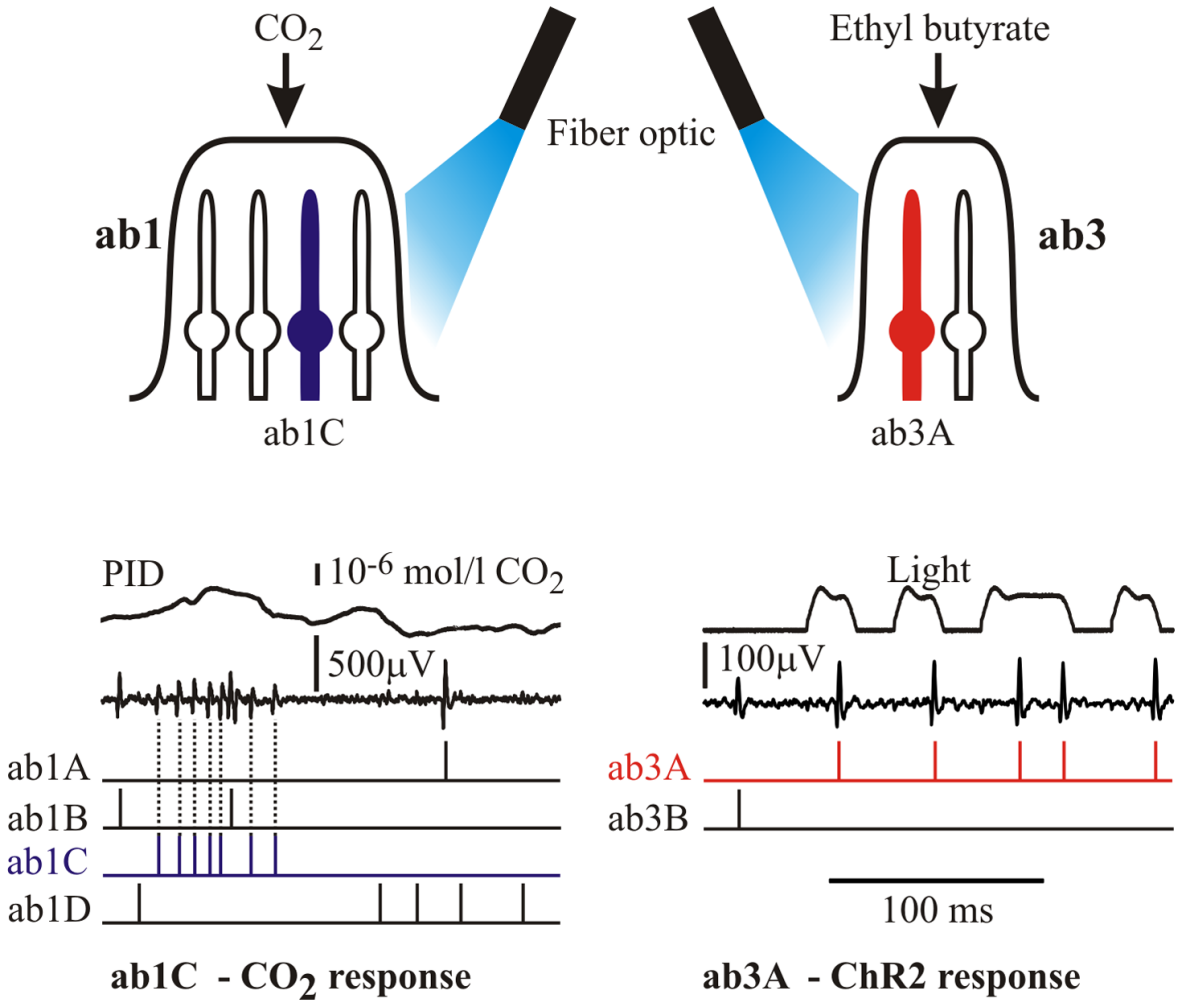
Three methods were used to stimulate basiconic sensilla. Upper: CO_2_ responses were recorded from ab1C neurons using the apparatus of Fig. 1. Ethyl butyrate stimulation of ab3A neurons used the same apparatus with a filter paper cartridge delivering the odorant into the air/propylene stream. Optical stimulation of ab1C and ab3A neurons containing channelrhodoposin-2 was performed by a high intensity blue light emitting diode via a fiber optic. Lower: Multiple action potential recordings were separated by cluster analysis. Raw recordings from an ab1 sensillum to CO_2_ (left) and ab3 sensillum to light (right) are shown together with the inputs (PID and light traces), and the separated action potential times.

All experiments were controlled by custom-written software via a personal computer and a data acquisition board (NI6035E, National Instruments, Austin, TX, USA). Binary M-sequences to drive the loudspeaker or the LED were both generated by the computer using a 33-bit binary shift register. The PID and recording electrode voltages were digitized via a 16-bit analog-to-digital converter and sampled at 0.2 ms intervals. Action potential signals were separated by a combined template matching and cluster analysis algorithm [21]. Single unit records were always visually checked against the original recordings to verify separation (Fig. 2).

Single unit times of occurrence were digitally filtered to a bandwidth of 0–100 Hz [26]. The PID voltage or LED current (input) and filtered action potential signal (output) were then re-sampled at 5 ms intervals. Sampled time domain data (20,000 input-output pairs) were transferred to the frequency domain using the fast Fourier transform [27] in segments of 1024 sample pairs. Frequency response functions between the PID voltage and action potentials were calculated by direct spectral estimation as complex (cosine and sine) functions of frequency, and plotted as Bode plots of phase and log amplitude versus log frequency [25]. Frequency response functions were fitted by minimizing the coherence-weighted square error between the complex data and a band-pass function:
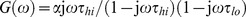
(1)where *G*(ω) is the frequency response function, j = (−1)^ ½^, ω is radial frequency, α is amplitude, and τ_hi_, τ_lo_ are time constants. The peak response of this function, *P*, occurs at ω = (τ_hi_ τ_lo_)^ −½^.

Coherence, γ^2^(ω), as a function of frequency [25], was calculated from the same data, and used to estimate the information capacity, *R*, of olfactory transduction [28]:
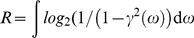
(2)


### Statistical Analysis

Tests for significant differences in means between pairs of distributions of fitted parameters were made using the non-parametric Mann-Whitney test. Statistical significance in the figures is indicated by asterisks: * p≤0.05, ** p≤0.01, *** p≤0.001.

## Results

The double pipet stimulating system (Fig. 1) was developed to overcome the difficulty of mixing CO_2_ and propylene tracer gas at high pressure. Since it relies on the principle of identical, simultaneous on-off switching of the two pipets, we checked the system by alternating the connections to the two pipets during identical experiments on the same fly. Results were always the same in either configuration, within the variability of single experiments. The concentration scale for CO_2_ was estimated from the calibrated sensitivity of the PID to propylene, assuming that the two pipets behaved identically.

**Figure 3:**
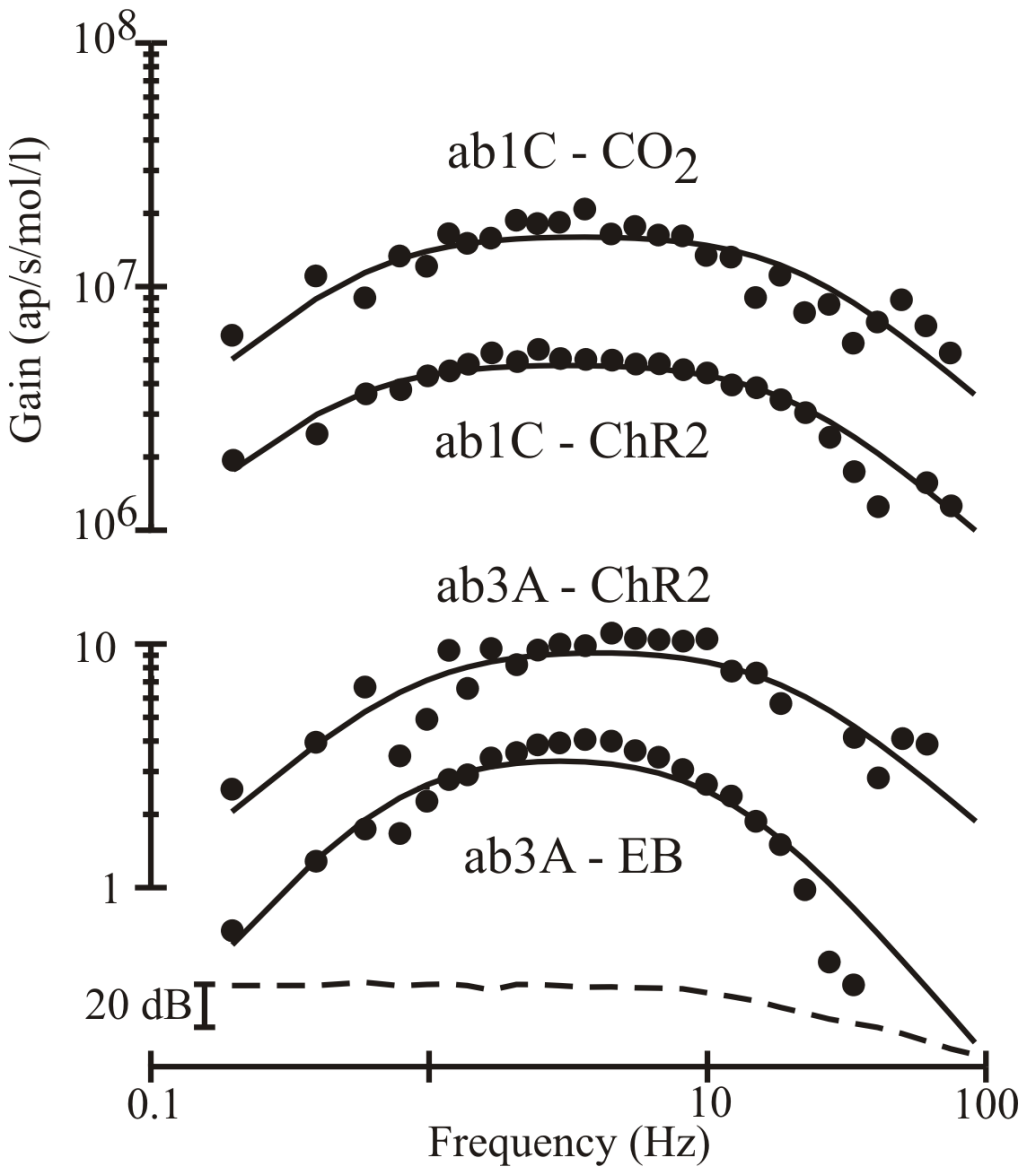
Gain portions of frequency response functions. Responses are shown for CO_2_ concentration, light intensity (ChR2), and Ethyl butyrate (EB) concentration (inputs) versus action potentials from ab1C and ab3A neurons (outputs). Recordings were 200 s duration. Experimental data (circles) were fitted by Equation 1 (solid lines). Gain units for CO_2_ stimulation were estimated from the calibrated sensitivity of the PID to propylene, assuming identical behavior of the two gases as they passed through the apparatus. Relative gain values are shown for ethyl butyrate and light stimulation, using the same logarithmic scaling as for CO_2_. Fitted time constants were (ab1C-CO_2_, ab1C-ChR2, ab3A-ChR2, ab3A-EB): τ_lo_ = 262 ms, 317 ms, 179 ms, 217 ms; τ_hi_ = 7.78 ms, 8.39 ms, 8.55 ms, 13.3 ms. Peak response to CO_2_ was 1.44×10^7^ ap/s/mol/l. The significantly higher value of τ_hi_ for ab3A-EB is visible in the steeper decline at high frequencies. Dashed line shows the input power spectrum for propylene concentration at the fly, acting as a surrogate tracer for CO_2_ or for ethyl butyrate.

The only known CO_2_ sensitive neuron in basiconic sensilla is ab1C [1], [2], so all CO_2_ experiments were performed on ab1 sensilla, followed by separation of the third largest action potentials by cluster analysis (Fig. 2). Frequency response functions were well fitted by the band-pass filter of Equation 1 (Fig. 3). Only the real gain portions of the frequency responses are shown, but the fitted parameters were always obtained from the complex gain functions that include phase information, and phase plots (not shown) were also well-fitted by Equation 1. Information capacity, *R*, was calculated from the coherence function, γ^2^(ω), of the same data in each case. The spectrum of the PID signal was approximately constant at low frequencies and declined less steeply than the neuron responses at high frequencies (Dashed line, Fig. 3).

We used ethyl butyrate stimulation of ab3A neurons via the same stimulation system to compare the dynamics of CO_2_ responses to a fruit odorant. This method provided reliable detection of single action potential unit responses to odorant [4]. Light stimulated responses to both neuron types were obtained from transgenic flies expressing ChR2 in the ab1C and ab3A neurons, respectively (Fig. 2). Frequency response functions for light stimulation were again well-fitted by Equation 1 (Fig. 3).

**Figure 4:**
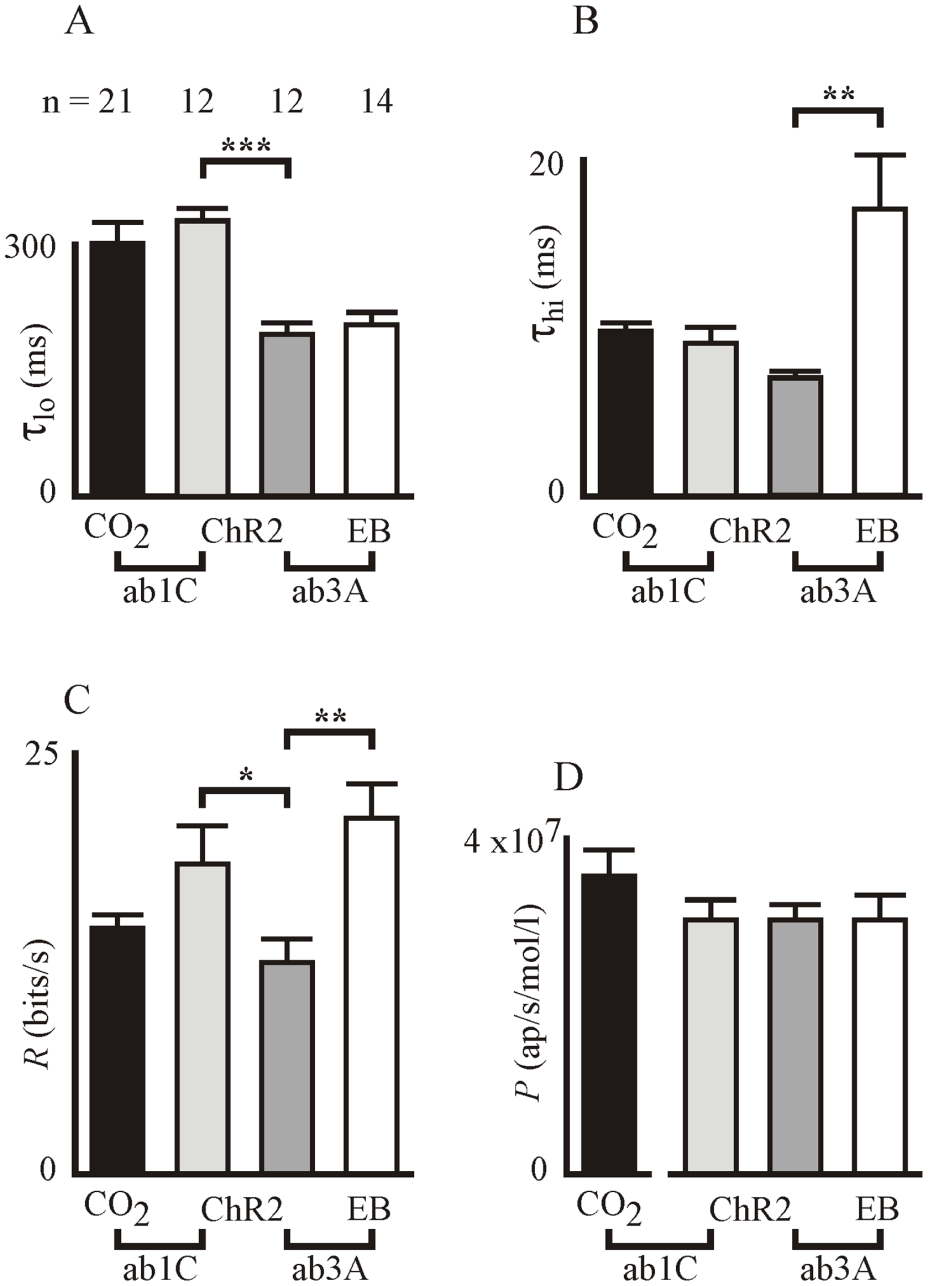
Summary of parameters for fitted frequency response functions. Responses between CO_2_ concentration and light intensity (inputs), and ab1C action potentials (outputs), or light intensity and ethyl butyrate (EB) concentration (inputs) and ab3A action potentials (outputs). A, B: Time constants τ_lo_ and τ_hi_, C: Information capacity, D: Peak response (ChR2 and EB are normalized values to show variance, only). Data are shown as means and standard errors for the indicated numbers of experiments (21, 12, 12 and 14 respectively). Data were compared by the Mann-Whitney nonparametric test for significant difference between means of distributions of independent samples. Asterisks indicate statistically significant differences at levels: p<0.05 (*), p<0.01 (**) and p<0.001 (***).

Fitted parameters of Equations1 and 2 were compared by the Mann-Whitney nonparametric test for significant difference between means (Fig. 4). Parameter τ_lo_ for CO_2_ stimulation of ab1C was 298±25 ms, not significantly different to τ_lo_ = 325±15 ms for light stimulation of the same neuron. In contrast, τ_lo_ for light stimulation of olfactory neuron ab3A was significantly lower than for ab1C (τ_lo_ = 191±13 ms), but not significantly different than for ethyl butyrate in ab3A (τ_lo_ = 202±15 ms).

The high frequency time constant, τ_hi_ = 9.68±0.52 ms for CO_2_ in ab1C was not significantly different than the parameter for light stimulation of ab1C or ab3A (τ_hi_ = 9.09±0.83, τ_hi_ = 6.94±0.41 respectively). However, this parameter was significantly larger for ethyl butyrate stimulation of ab3A (τ_hi_ = 16.9±3.22 ms).

Information capacity, *R* (Fig. 4C), was significantly higher for light stimulation of ab1C (18.3±2.2 bit/s) than ab3A (12.5±1.4 bits/s) but not significantly different than for CO_2_ stimulation of the same neuron (14.5±0.8 bits/s). In contrast, ethyl butyrate stimulation of ab3A gave a significantly higher value of *R* (21.0±2.05 bits/s) than light stimulation. Peak response, *P*, of the ab1C neuron to CO_2_ was 3.52±0.31 ap/s/mol/l. Peak responses for light stimulation of ab1C and ab3A, as well as ethyl butyrate stimulation of ab3A could not be calibrated because neither the evaporation rate of odorant or the number of photons reaching the ab3A neuron were known. The mean and standard errors for these parameters are shown at similar scale to *P* for CO_2_, but only to illustrate their relative variabilities (Fig. 4D).

## Discussion

### The Linear Approach, Stimulus and Response

Systems analysis requires that input and output signals be well characterized. The olfactory approach used here, by always measuring surrogate tracer gas as close as possible to the receptor [14], [18], [29], aims to eliminate the need to accurately control odorant concentration in space or time, since the major requirements for direct spectral analysis are only that the input signal be known, and have wide bandwidth. In fact, this approach has been used successfully before with turbulent stimulation [29], and for characterizing different stimulation geometries and flow rates [30]. However, the laminar flow method increases input accuracy by creating a stimulus that is approximately constant with position relative to the tube mouth, and a linear function of driving signal [23], [24]. The optical stimulus was measured as current through the light emitting diode, which is well characterized as proportional to light output.

Encoding of action potentials from an applied membrane current is an inherently nonlinear process, in time and amplitude [31], and many physiological systems, including neurons, have additional nonlinear dynamic properties [32]. Nonlinear dynamic system response depends critically on the nature of the stimulus inputs, so although Gaussian white noise has been used extensively for linear and nonlinear systems analysis [32]–[34] there is increasing use of stimuli that more closely resemble natural stimuli received by an animal [35], [36]. Measures of sensory system performance, particularly information transmission, are also developing rapidly beyond simple signal-to-noise estimates, with increasing emphasis on quantitation of entropy within signals, and mutual information between input and output signals [36].

While a full description of sensory transduction and information encoding by insect olfactory sensilla will eventually require more complete exploration of their stimulus space and nonlinear dynamics, the present experiments were designed with the more limited goals of comparing the dynamics of CO_2_ to fruit odorant transduction, and separating the dynamic contributions of action potential encoding from the earlier steps of olfactory transduction. Linear systems analysis, which has been widely applied to spiking neurons [33], [34], provided a useful first approximation to these questions. Insect olfactory sensilla have approximately linear antennograms with non-saturating stimuli [12], [14], [37], although antennograms, may only represent the first stage of transduction to receptor current [38], and frequency response functions of single unit recordings were well fitted by cascades of linear filters followed by mildly nonlinear static components [18], [39]. Supporting this, *Drosophila* olfactory sensilla experiments using laminar flow stimulation had coherence approaching unity over the region of peak frequency response [40], indicating approximately linear behavior.

### Interpretation of the Two Time Constants

The two time constants, τ_lo_ and τ_hi_, are inversely proportional to the lower and upper frequencies at which the system’s output begins to decrease as the stimulus frequency is varied. A system that continued to respond to a constant (zero frequency) stimulus would have infinite τ_lo_, so a lower value reflects the rate of adaptation to a constant stimulus. In contrast, τ_hi_, defines the upper frequency range of the system’s ability to respond.

While there were significant differences among the time constants obtained from the four stimulation methods, the relative similarity of the frequency response functions is striking (Fig. 3). The most parsimonious explanation for this similarity is that the dynamic responses reflect similar physiological processes in each case.

ChR2 stimulation causes direct depolarization of a neuron membrane [41], eliminating odorant transduction and any associated second messenger systems from the dynamic response. This leaves the dynamic responses of action potential encoding by voltage-activated ion channels, and any associated processes. Stimulation of ab1C by CO_2_ or light produced statistically identical values of both time constants, indicating that the dynamic properties of CO_2_ responses are limited by processes following the depolarization produced by the CO_2_ transduction cascade.

The results for ab3A sensilla were more complex because the high frequency parameter, τ_hi_, depended strongly on the stimulation method. One hypothesis is that the relatively large ethyl butyrate molecules were slower to enter the sensillum, dissolve and move to the sensory neuron membrane than the transduction, depolarization and action potential firing. This hypothesis would also require that CO_2_ can accomplish these steps more rapidly. Another possibility is that the transduction process itself is significantly different between CO_2_ and ethyl butyrate.

A significant difference was detected between the low frequency responses of ab1C and ab3A neurons to light stimulation (Fig. 4). Since the stimulus and transduction mechanisms were identical in each case, this indicates that the dynamic properties of action potential encoding are different in the two neurons. A range of ionic mechanisms have been proposed to explain deviations in action potential encoder responses at low or high frequencies, including sodium or potassium channel activation or inactivation, calcium-mediated feedback, and electrogenic sodium pumping [34]. Genetic reduction of voltage-activated sodium currents caused a drop in the plateau phase of responses to pulsed odor stimulation of *Drosophila* olfactory receptor neurons [39], supporting the role of action potential production in limiting the low frequency response. If sodium channels indeed control the low frequency dynamics, the differences between ab1C and ab3A neurons could depend on activation or inactivation parameters, as has been shown in some paired mechanoreceptor neurons [42].

When comparing the experiments with light and chemical stimulation it is important to note that similar linear measurements could result from different, but complementary nonlinear processes. Nevertheless, the simplest explanation for the similarities remains that action potential encoding dominates the low frequency responses of these neurons. Some caution is also necessary in interpreting ethyl butyrate data because the paper cartridge stimulation method is inherently less reliable than the more accurately controlled light and CO_2_ experiments. More detailed experimental and analytical approaches may be useful to clarify these issues in the future.

Based on these data, *Drosophila* antennae are able to detect changes in CO_2_ concentration from below 0.1 Hz to more than 100 Hz. This agrees with, and extends previous tests of ab1C responses to pulsed CO_2_, which found reliable responses at a rate of 10 pulses per second [9]. That study also found no significant adaptation to repeated 500 ms pulses, in agreement with the low frequency range seen here. However, the form of frequency response (Equation 1; Fig. 3) would indicate that the neuron eventually ceases responding to a constant level of CO_2_ if the relationship holds to zero frequency. Pulse responses of neuron ab1C to CO_2_ were also reported to be more reliable than responses of ab1A to ethyl acetate [9], which again agrees with our finding that CO_2_ stimulation gave a significantly wider bandwidth response than ethyl butyrate stimulation.

Any effects of odorant diffusion rate to sensilla should have been eliminated in our experiments because we detected the tracer gas at the sensillum itself. Odorants vary significantly in their dynamic access to sensilla but differences in response rise time persist after eliminating such effects [12], [43]. This agrees with our finding that the high frequency time constant varied with the type of odorant. It has been suggested that the ability of a chemoreceptor to respond to concentration changes in time implies a mechanism for terminating the response to each detected molecule, by processes such as receptor inactivation or odorant binding [12], and such odorant flux has been included in models of pheromone component detection [15]. The dynamics of *Drosophila* olfaction are also relatively independent of concentration [43], supporting the approximately linear relationship between input and output that we observed here and previously [21].

### Information Capacity and CO_2_ Sensitivity

Information capacity values ranged from 12–21 bits/s. There were significant differences between the two neuron types with light stimulation, and between light and ethyl butyrate stimulation of ab3A neurons. Since the responses were approximately linear, these data were probably dominated by signal-to-noise levels in the neurons, but it is impossible to estimate actual signal and noise values because the signal levels produced by the three types of stimulation at the receptor current stage are unknown. These information capacity values were similar to the range of approximately 10–20 Bits/s reported previously for a series of fruit odors in *Drosophila* [21], but they were significantly lower than those reported for many other spiking sensory receptors [19]. The low values are most probably due to the frequency ranges of the responses, which were much smaller than many mechano- and photo-receptors [19], because the integration in Equation 2 is over all frequencies. Nevertheless, the information capacity values indicate that the antennal CO_2_ detection system has similar reliability to those for fruit odors.

The numbers of CO_2_ molecules being transduced by a basiconic sensillum will depend on the fluid dynamics and boundary layer conditions at the antennal surface, as well as the number and size of the pores in the sensillum wall. A simple estimate of the available numbers can be made by assuming that the volume of gas surrounding a sensillum is similar to the sensillum itself. For a large basiconic sensillum in *Drosophila* this is about 25 µm^3^ [44]. At standard temperature and pressure, the mean sensitivity of 3.52±0.31 ap/s/mol/l for CO_2_ would correspond to a change of one action potential per second for each increase of 5×10^9^ CO_2_ molecules in the surrounding volume.

Many important questions remain about CO_2_ detection by ab1C neurons. It is still unknown if the gas acts in its native form or after conversion to bicarbonate, or how the two odorant receptors function together [1]. Our results indicate that primary CO_2_ sensation is broadly comparable to other odors sensed by basiconic sensilla. They also indicate that dynamic characteristics of action potential encoding vary between identified neurons and probably make major contributions to overall sensory dynamic responses.
